# A novel sequestrate species from Mexico: *Aroramyces guanajuatensis* sp. nov. (Hysterangiaceae, Hysterangiales)

**DOI:** 10.3897/mycokeys.61.36444

**Published:** 2019-12-11

**Authors:** Rafael Peña-Ramírez, Zai-Wei Ge, Rigoberto Gaitán-Hernández, César Ramiro Martínez-González, Gonzalo Guevara-Guerrero

**Affiliations:** 1 Instituto Tecnológico de Cd. Victoria, Av. Portes Gil 1301 Pte. C.P. 87010, Cd. Victoria Tam, Mexico; 2 Kunming Institute of Botany, Chinese Academy of Sciences, 132, Lanhei Road, Kunming 650201, China; 3 Instituto de Ecología, A.C., Carretera antigua a Coatepec # 351, El Haya, C.P. 91070, Xalapa, Veracruz, Mexico; 4 Universidad Nacional Autónoma de México, Profesor de Asignatura B, Departamento de Biología, Facultad de Ciencias Ciudad Universitaria, Delegación Coyoacán, 04510, Ciudad de México, Mexico

**Keywords:** Truffle, truffle-like, sequestrate fungi, hypogeous fungi

## Abstract

Knowledge of sequestrate Hysterangiaceae fungi in Mexico is very limited. In the present study, a new member of the family, *Aroramyces guanajuatensis***sp. nov.**, is described. This speciesis closely related to *A. balanosporus*, but differs from the latter in possessing a tomentose peridium 165–240 µm thick, with occasional large terminal hyphae up to 170 µm, a variable mesocutis (isodiametric to angular), and distinct bright yellowish subcutis. In contrast, *A. balanosporus* possesses a fibrillose peridial surface with shorter hyphae, a peridium 200–450 µm thick, and a mainly hyaline isodiametric mesocutis with a slightly wider subcutis. The phylogenetic analysis of the LSU gene separated *A. guanajuatensis* from *A. balanosporus* with a Bayesian posterior probability (PP) = 1. This is the third *Aroramyces* species described for the American continent.

## Introduction

*Aroramyces* Castellano and Verbeken was coined to settle *Hymenogaster radiatus* (Lloyd, 1925) and *Hysterangium gelatinosporum* (Cribb, 1957) from two different genera (Castellano et al. 2000). Phylogenetic analysis places *Aroramyces* near, but different to *Hysterangium* (Hosaka et al. 2006, 2008). *Aroramyces* is characterized by its unique combination of a brown gleba, spiny spores with distinctly inflated utricles, gelatinized gleba, and basidiome with a tomentose surface with numerous soil particles adhering to all sides. At present, there are four species in this group (Kirk 2018): *Aroramyces radiatus* (Lloyd) Castellano, Verbeken & Walleyn, *A. gelatinosporus* (J.W. Cribb) Castellano (Castellano et al. 2000), *A. balanosporus* G. Guevara & Castellano, and *A. herrerae* G. Guevara, Gomez-Reyes & Castellano (Guevara-Guerrero et al. 2016). *Aroramyces guanajuatensis* was discovered during a survey aiming to document the fugal diversity in Guanajuato, Mexico. It is therefore determined that the number of *Aroramyces* species described in the American continent is now three.

## Materials and methods

### Sampling and morphological characterization

The collections were discovered with a cultivator, digging around trees up to a depth of 15 cm. All encountered fruiting bodies were photographed fresh and then dried at 50 °C. The chosen material was cut by hand and rehydrated with 5% KOH for morphological studies. Thirty spores were measured. Peridial slices were made and observed under optical microscopy (Castellano et al. 1989). For scanning electron microscopy pictures (JSM5600LV, JOEL, Tokyo, Japan), the spores were coated with gold and palladium. Voucher collections are deposited at José Castillo Tovar (**ITCV**) Herbarium, Instituto Tecnólogico de Ciudad Victoria, Mexico.

### DNA extraction, amplification, sequencing and phylogenetic analyses

Genomic DNA was obtained with CTAB (Martínez-González et al. 2017)) or using Fungal DNA extraction Kit (Bio Teke Corporation, China) from 2–3 mg of dry tissue. DNA quantification was performed with Nanodrop (Thermo, USA). Each sample was diluted to 20 ng/uL for PCR amplification. LR0R and LR5 primers were used to amplify the LSU gene (Vilgalys and Hester 1990). The PCR reaction contained the following: enzyme buffer 1×, *Taq* DNA polymerase, 0.8 mM deoxynucleoside triphosphates (0.2 mM each), 100 ng DNA, 20 pmol of each primer, and 2 units of Go*Taq* DNA (Promega, USA), with a final volume of 15 µL. The amplification program was run as follows: denaturalization at 96 °C for 2 min, 35 cycles of denaturalization at 94 °C for 1 min, annealing at 57 °C for 1 min, polymerization at 72 °C for 1 min, and final elongation at 72 °C for 5 min. All PCR reactions were carried out in a Peltier Thermal Cycler PTC-200 (BIORAD, Mexico). The PCR products were verified by agarose gel electrophoresis run for 1 h at 95 V cm^-3^ in 1.5% agarose and 1× TAE buffer (Tris Acetate-EDTA). The products were then dyed with GelRed (Biotium, USA) and viewed in a transilluminator (Infinity 3000 Vilber, Loumat, Germany). Finally, the products were purified using the ExoSap Kit (Affymetrix, USA) according to the manufacturer’s instructions and were prepared for the sequencing reaction using the BigDye Terminator Cycle Sequencing Kit v. 3.1 (Applied BioSystems).

Sequencing was carried out in a genetic analyzer (Model 3130XL, Applied BioSystems, USA) at the Biology Institute of the National Autonomous University of Mexico (UNAM). The sequences of both strains of each sample were analyzed, edited, and assembled using BioEdit v. 1.0.5 (Hall 1999) to create consensus sequences. The consensus sequences were compared with those in the GenBank database of the National Center for Biotechnology Information (NCBI) using the BLASTN 2.2.19 tool (Zhang et al. 2000). The LSU region was aligned using the online version of MAFFT v. 7 (Katoh et al. 2002, 2017; Katoh and Standley 2013). The alignment was revised in PhyDE (Müller et al. 2005), and small manual adjustments were then made to maximize the similarity between characters. The matrix was composed of 30 taxa (640 characters) (Table 1). *Ramaria gelatinosa* (access number AF213091) was used as the outgroup. The phylogeny was performed using Bayesian inference in MrBayes v. 3.2.6 64× (Huelsenbeck and Ronquist 2001). The information block matrix included two independent runs of the MC3 chains for ten million generations (standard deviation ≤ 0.01); the reversible-jump strategy was used (Huelsenbeck et al. 2004). An evolutionary model was used, so a proportion of invariable sites were designated, and the other proportion came from a gamma distribution (invgamma). The convergence of chains was visualized in Tracer v. 1 (Rambaut et al. 2014). The phylogram of maximum credibility for the clades was recovered in TreeAnotator v. 1.8 (Bouckaert et al. 2014) based on the burning of 2.5 million trees.

**Table 1.** List of NCBI accession numbers for LSU and ITS sequences of *Aroramyces guanajuatensis*.

Herbarium number	LSU NCBI number	ITS NCBI number
ITCV 1689	MK761021	MN392935
ITCV 1691	MK761022	MN392936
ITCV 1694	MK761023	MN392937
ITCV 1711	MK761024	MN392938
ITCV 1610	MK761025	MN392939
ITCV 1610	MK811035	–
ITCV 1613	MK761026	MN392940
ITCV 1613	MK811036	–
ITCV 1729	MK761027	MN392941
ITCV 1731	MK761028	MN392942
ITCV 1734	MK761029	MN392943
ITCV 1738	MK761030	MN392944
ITCV 1739	MK761031	MN392945
ITCV 1741	MK761032	MN392946

## Results

### Molecular analyses

**Figure 1.**
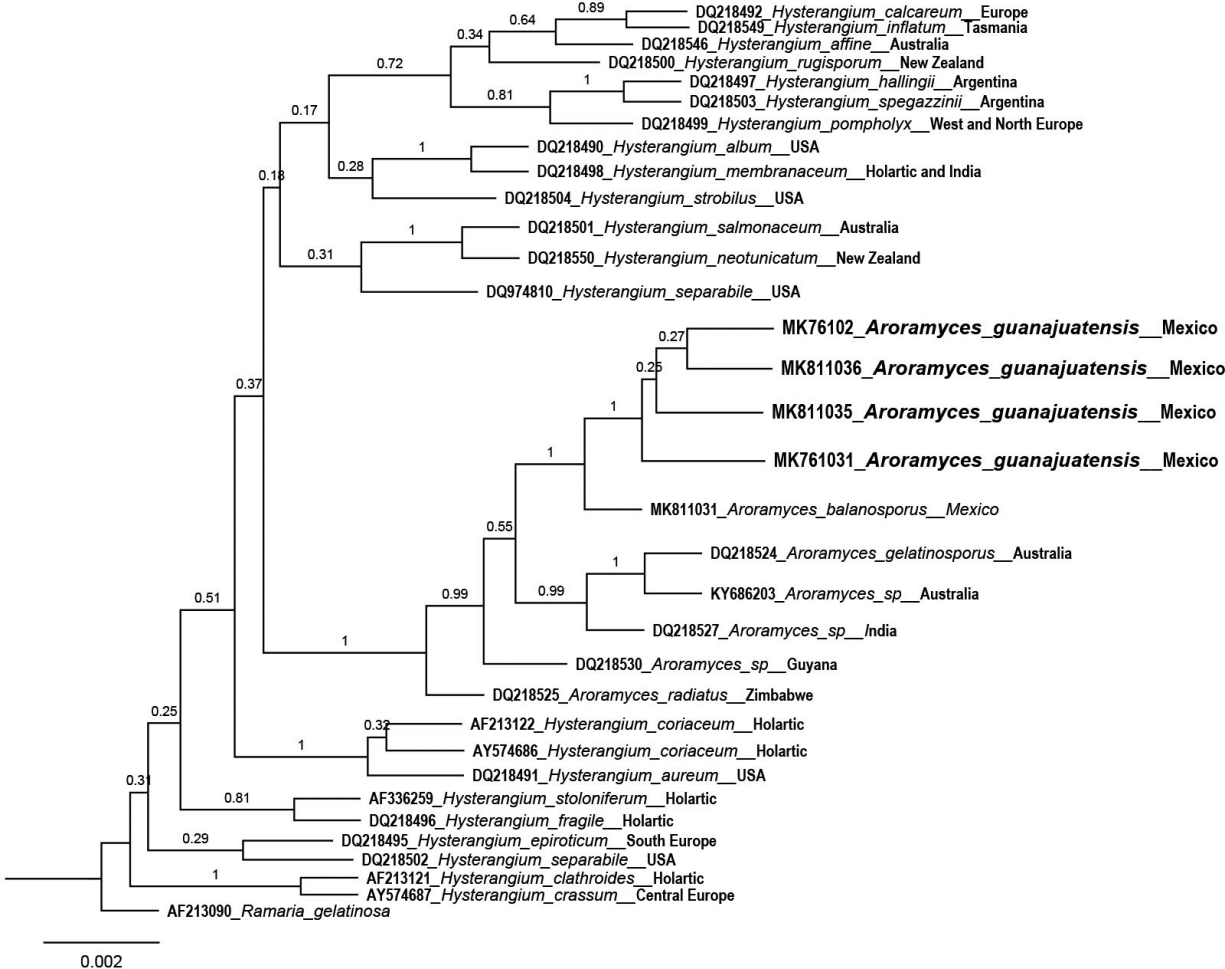
Maximum probability phylogram of clades obtained with Bayesian inference. The posterior probabilities for each clade are shown on the branches. The accession numbers in the sequence labels indicate the GenBank accession numbers.

ITS and LSU sequences of 12 samples *of A. guanajuatensis* were obtained (Table 1). ITS and LSU sequences are respectively identical. Based on this, only four sequences were selected for phylogenetic analysis. Then after, alignment was performed with 6 sequences of *Aroramyces* and 22 sequences of *Hysterangium* (Table 2). Phylogenetic results were as follows: According to the Bayesian analysis, after 10 million generations, 25% trees were discarder as the burn-in. The standard deviation between the chains stabilized at 0.002, indicating that MC3 reached a stationary phase. To confirm that the sample size was enough, the “parameter” file was analyzed using Tracer v. 1.6 (Rambaut et al. 2014), verifying that all parameters had an estimated sample size above 1,500. The subsequent probabilities (SP) were estimated based on the strict consensus rule produced by MrBayes and indicated on the maximum credibility clade tree. The Bayesian inference analysis recovered *A. guanajuatensis* as a monophyletic group, with a posterior probability of 1. (Fig. 1). *Ramaria gelatinosa* was used to root the tree. *Aroramyces balanosporus* and *A. guanajuatensis* showed the closest relationship but were branched with a probability of 1 and a dissimilarity of 2.19, supporting the existence of a new taxon. The *Hysterangium* species segregated and formed two different branches.

**Table 2.** List of *Aroramyces* and *Hysterangium* species, GenBank accession numbers, and references for LSU sequences used in the phylogenetic analysis. Sequences of new taxon are in bold.

Species	GenBank	Reference
*Aroramyces balanosporus* G. Guevara & Castellano	MK811031	This paper
***A. guanajuatensis***	MK761024	This paper
	MK811036	This paper
	MK811035	This paper
	MK761031	This paper
*A. gelatinosporus* (J.W. Cribb) Castellano	DQ218524	Hosaka et al. 2008
*A. radiatus* (Lloyd) Castellano, Verbeken & Walleyn	DQ218525	Hosaka et al. 2008
*Aroramyces* sp.	KY686203	Nuske & Abell unpublished
	DQ218527	Hosaka et al. 2008
	DQ218530	Hosaka et al. 2008
*Hysterangium affine* Mase & Rodway	DQ218546	Hosaka et al. 2008
*H. album* Zeller & C.W. Dodge	DQ218490	Hosaka et al. 2008
*H. aureum* Zeller	DQ218491	Hosaka et al. 2008
*H. calcareum* R. Hesse	DQ218492	Hosaka et al. 2008
*H. clathroides* Vittad	AF213121	Humpert et al. unpublished
*H. coriaceum* R. Hesse	AF213122	Humpert et al. unpublished
	AY574686	Giachini et al. 2010
*H. crassum* (Tul & C. Tul) E, Fisch	AY574687	Giachini et al. 2010
*H. epiroticum* Pacioni	DQ218495	Hosaka et al. 2008
*H. fragile* Vittad	DQ218496	Hosaka et al. 2008
*H. hallingi* Castellano & J.J. Muchovej	DQ218497	Hosaka et al. 2008
*H. inflatun* Rodway	DQ218549	Hosaka et al. 2008
*H. membranaceum* Vittad	DQ218498	Hosaka et al. 2008
*H. neotunicatun* Castellano & Beever	DQ218550	Hosaka et al. 2008
*H. pompholyx* Tul. & C. Tul.	DQ218499	Hosaka et al. 2008
*H. rugisporum* Castellano & Beever	DQ218500	Hosaka et al. 2008
*H. salmonaceum* G.W. Beaton, Pegler & T.W.K. Young	DQ218501	Hosaka et al. 2008
*H. separabile* Zeller	DQ974810	Smith et al. 2007
	DQ218502	Hosaka et al. 2008
*H. spegazzinii* Castellano & J.J. Muchovej	DQ218503	Hosaka et al. 2006
*H. stoloniferum* Tul. & C. Tul.	AF336259	Binder and Bresinsky 2002
*H. strobilus* Zeller & C.W. Dodge	DQ218504	Hosaka et al. 2006

### Taxonomy

#### Aroramyces guanajuatensis

Taxon classificationAnimaliaHysterangialesHysterangiaceae

Peña-Ramírez, Guevara-Guerrero, Z. W. Ge & Martínez-González sp. nov.

3F46ADC5-EDF7-57F8-8EF8-CFB4304059F2

30329

Figures 2 , 3 , 4

##### Type.

MEXICO. State of Guanajuato, municipality of Guanajuato, Cuenca de la Esperanza Protected Natural Area, 7 November. 2016Peña-Ramírez 108 (Holotype: ITCV 1613).

##### Diagnosis.

*Aroramyces guanajuatensis* is characterized by a peridium 167–240 µm thick, of cotton-like hyphae, up to 170 µm, long, variably structured mesocutis, yellowish subcutis, spores with irregular and inflated utricle.

**Figure 2.**
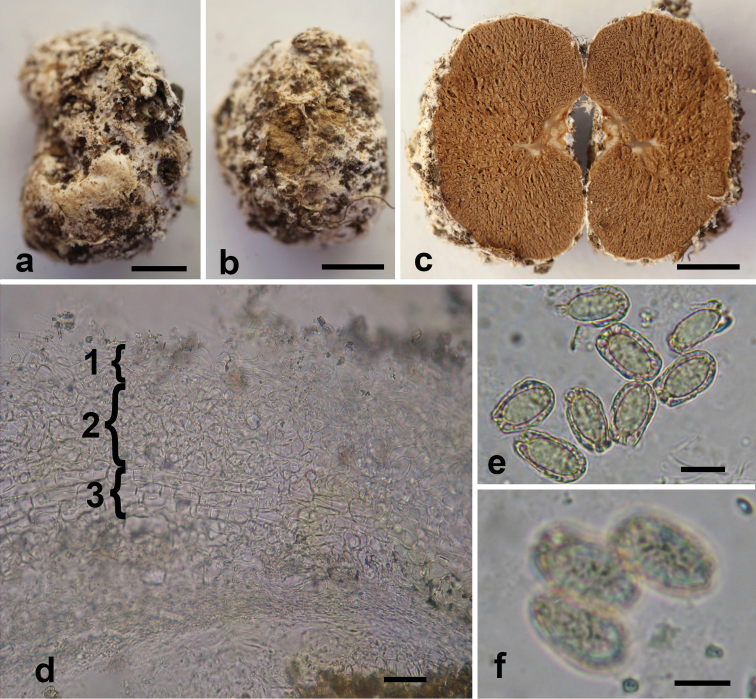
**a–f***Aroramyces guanajuatensis* (holotype ITCV 1613) **a, b** basidiome showing the peridial surface **c** basidiome in cross-section showing glebal surface **d** cross-section of peridium showing three-layered peridium (1 epicutis, 2 mesocutis, 3 subcutis) at 400× **e–f** basidiospore 1000× **f** irregular crest contained within an inflated utricle. Scale bars: 0.5 cm (**a–c, f**), 20 µm (**d**), 5 µm (**e**).

**Figure 3.**
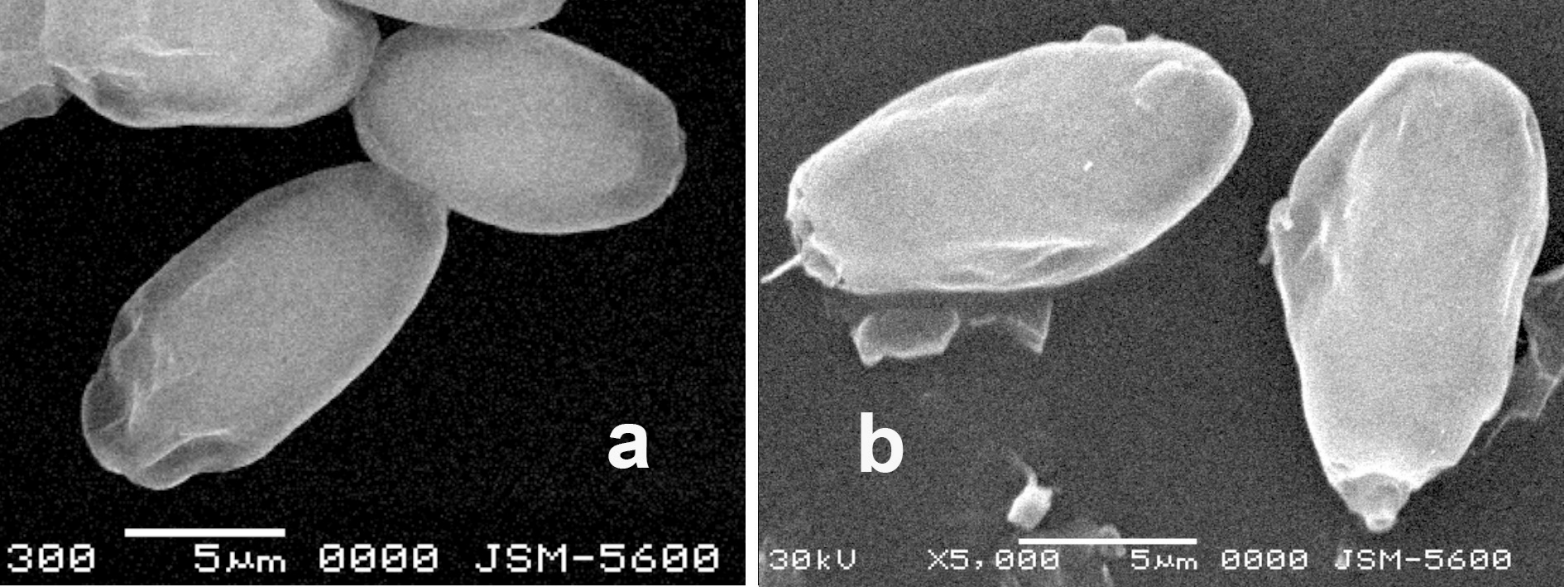
Electronic photomicrograph of basidiospores **a***Aroramyces balanosporus* (ITCV 848) and **b***Aroramyces guanajuatensis* (ITCV 1739). Scale bars: 5 µm.

##### Etymology.

"*guanajuatensis*" in reference to the site (Guanajuato state) where the new taxon was discovered.

##### Description.

Basidiome 4.4–17×3.9–13.5×3.2–11.2 mm, globose or subglobose to irregular, sometimes compressed when growing together. Peridial surface white, pale brown, fibrillose or tomentose, often with cotton-like patches of white hyphae encompassing some debris soil, stones, leaves, and roots. Peridium Separable and fragile, exposing portions of the gleba. < 0.5 mm thick, mostly hyaline, outer portion pale brown, with a dark ring next to the gleba. Gleba brown, trama gelatinized, locules irregularly shaped, columella dendroid, translucent gray. Odor fungoid; taste not recorded. Basidiomata hard wen dried.

Peridium three layered, 165–240 µm thick. Epicutis 7.5–22.5 µm thick of hyaline to reddish brown, thin-walled, interwoven to repent or erect hyphae, 2–9 µm wide, forming scattered caespitose groups of erect, branched, setal hyphae up to 170 µm long, with abundant crystalline structures adherent on hyphal walls, clamp connections present. Mesocutis 55–105 µm thick, abundant hyaline, isodiametric, globose to subglobose, angular pseudoparenchyma like cells. 4–35×3–24 µm, also with some irregularly shaped, interwoven hyphae, 3–11 µm wide. walls 1–2 µm µm wide, clamp connection absent. Subcutis 22.5–95 µm thick, of interwoven prostrate hyphae, 3–4 µm broad, with scattered large pseudoparenchyma like cells up to 37.5×30 µm, clamp connection absent.

Trama of hyaline, interwoven hyphae 4 µm wide, embedded in a gelatinized matrix, clamp connections present.

Basidia fusoid to clavate, hyaline, 14–48 × 9–12 µm, mean = 32.5 × 10.3 µm, wall 1 µm thick. Basidiospores ellipsoid to broadly ellipsoid, symmetrical, hyaline to pale brown, slightly reddish in KOH, pale brown in mass, excluding utricle 9–13 × 6–7 µm long, mean = 11 × 6.1 µm, Q range = 1.5–2.17, Q mean = 1.8; with utricle 12–17 × 7–10 µm long, mean = 14.47 × 8.27 µm, Q = 1.5–2.13, mean = 1.76. Ornamentation of irregular crest contained within an inflated utricle, hilar appendage in cross-section appears rectangular, 1–3 × 4–6 µm, mean = 1.97 × 5.2 µm. Apex obtuse. Utricle inflated up to 3 µm from spore wall, mean = 1.43 µm, occasionally the utricle is asymmetrically inflated.

**Figure 4.**
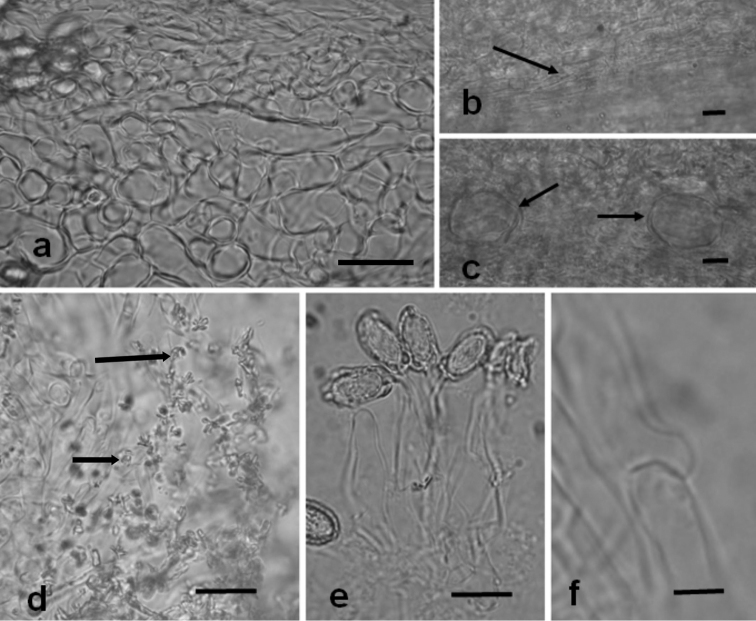
**a–f***Aroramyces guanajuatensis* (holotype ITCV 1613) **a–c** Cross-section of peridium **a** epicutis of hyphae postrate cells and angular pseudoparenchyma like mesocutis **b** arrow head showing postrate cell subcutis **c** arrow head showing large cell scattered in subcutis **d** arrow head showing epicutis hyphae with attached crystals **e** basidia and basidioles **f** epicutis clamp connection. Scale bar: 10 µm (**a–c, e**), 25 µm (**d**), 5 µm (**f**).

##### Distribution, habit and ecology.

MEXICO, state of Guanajuato. Cuenca de la Esperanza Protected Natural Area. Hypogeous, under *Quercus* spp. at 2530 m. 21°03.87'N, 101°13.193'W. October to December. The March collection was dried in situ.

##### Additional material examined.

Mexico, state of Guanajuato, Cuenca de la Esperanza Protected Natural Area: 21°04.075'N, 101°13.193'W 2500 m, 26 October 2016, Peña-Ramírez 102, paratype (ITCV 1610). 21°03891'N, 101°13.531'W 2457 m, 5 October 2016, Peña-Ramírez 70 (ITCV 1689). 21°03.891'N, 101°13.533'W, 2464 m, 5 October 2016, Peña-Ramírez 72 (ITCV 1691). 21°03.88'N, 101°13.561'W, 2471 m, 5 October 2016, Peña-Ramírez 75 (ITCV 1694). 21°03.85'N, 101°13.278'W. 2533 m, 19 October 2016 Peña-Ramírez 92 (ITCV 1711). 21°03.741'N, 101°13.461'W. 2500 m, 14 November 2016, Peña-Ramírez 110 (ITCV 1729). 21°03.901'N, 101°13.551'W. 22 December 2016, Peña-Ramírez 119 (ITCV 1738). 21°03.905'N, 101°13.018'W. 2458 m. 22 December 2016, Peña-Ramírez 120 (ITCV 1739). 21°04.045'N, 101°13.018'W. 2508 m, 6 March 2017, Peña-Ramírez 122 (ITCV 1741).

## Discussion

In the Bayesian inference analysis, Various *Aroramyces* nest together along with undescribed species mentioned in Nuske & Abell (unpublished) and Hosaka et al. (2008). The clade of the genus *Aroramyces* segregated between two clades that group species of *Hysterangium*. The close relationship of *Aroramyces* to *Hysterangium* in our study agrees with Hosaka et al. (2006, 2008). *Aroramyces balanosporus* and *A. guanajuatensis* are closely related but are morphologically and molecularly distinct (Figure 1). Although, the objective of the current assignment is not inferring the phylogenetic relationships in Hysterangiaceae, the result clearly supports *Aroramyces guanajuatensis* to be an independent species within the genus *Aroramyces* with a posterior probability of 1.

The hilar appendage is larger in *Aroramyces guanajuatensis* compared to *A. balanosporus*. The isodiametric mesocutis cells of *A. guanajuatensis* differ from the variously shaped cells in the mesocutis of *A. balanosporus*. The asymmetric utricle of *Aroramyces herrerae* up to 6 µm wide whereas the asymmetric utricle of *A. guanajuatensis* rarely reaches 3 µm wide. Mexican *Aroramyces* are associated with *Quercus* spp. Interestingly *A. radiatus* from Africa has smaller spores, 10–12(–13.5) × 6–7(–8) μm, and is associated with *Brachystegia spiciformis* (Caesalpinioideae) and *Upaca* sp. (Euphorbiaceae). *Aroramyces gelatinosporus* from Australia has similar sized spores but a single-layered peridium and is associated with *Eucalyptus* spp. (Myrtaceae) (Castellano et al. 2000; Lloyd 1925).

The collections were discovered in the Cuenca de la Esperanza Protected Natural Area in Guanajuato, Mexico, located north of Michoacán and east of Jalisco. The presence of unidentified species in this region highlights the importance of this protected natural area and as an area to search for additional new fungal taxa.

## Supplementary Material

XML Treatment for Aroramyces guanajuatensis
